# Parental Smoking and Smoking Status of Japanese Dental Hygiene Students: A Pilot Survey at a Dental Hygiene School in Japan

**DOI:** 10.3390/ijerph6010321

**Published:** 2008-01-19

**Authors:** Toru Naito, Koichi Miyaki, Mariko Naito, Masahiro Yoneda, Nao Suzuki, Takao Hirofuji, Takeo Nakayama

**Affiliations:** 1 Department of General Dentistry, Fukuoka Dental College, Tamura 2–15–1, Fukuoka 814–0193, Japan; E-mails: yoneda@college.fdcnet.ac.jp (M. Y.); naojsz@college.fdcnet.ac.jp (N. S.); hirofuji@college.fdcnet.ac.jp (T. H.); 2 Department of Health Informatics, School of Public Health, Kyoto University Graduate School of Medicine, Yoshida-Konoe, Kyoto 606–8501, Japan; E-mails: miyaki@pbh.med.kyoto-u.ac.jp (K. M.); nakayama@pbh.med.kyoto-u.ac.jp (T. N.); 3 Department of Preventive Medicine, Biostatistics and Medical Decision Making, Nagoya University Graduate School of Medicine, Tsurumai 65, Nagoya 466–8550, Japan; E-mail: mnaito@med.nagoya-u.ac.jp (M. N.)

**Keywords:** Smoking, smoking prevention, questionnaire survey, decision tree analysis

## Abstract

This study aimed to determine the frequency of smoking and to explore factors associated with the smoking habits of female students at a dental hygiene school in Japan. Questionnaires regarding cigarette smoking were given to 168 female students. The response rate was 97.6%. The prevalence of smoking, including current and occasional smokers, was 20.3%. Among family members, only the smoking status of their mother significantly influenced the smoking status of the students. The odds ratio for smoking among dental hygiene students whose mothers were smokers in comparison to students whose mothers were not smokers was 5.1 (95% confidence interval 2.1–12.2, *p*=0.000). Decision tree analysis showed that the smoking habit of dental hygiene students was correlated with their mothers’ smoking history, as well as the smoking status of junior high school teachers, the smoking habits of close friends and a history of participating in a smoking prevention program when in elementary school. The increased smoking rate of young females, including dental hygienists, is a growing problem in Japan. The smoking statuses of dental hygiene students might be closely influenced by their mothers’ smoking status.

## 1. Introduction

It is well known that cigarette smoking is a global problem [1, 2]. Especially for oral healthcare professionals, the recognition that oral cancer and periodontal disease are adverse health outcomes of smoking is particularly important; therefore, reducing the use of tobacco products has been identified as a major target for health promotion activities of the World Health Organization policy of ‘Health for all in the 21st century’ [3].

Dentistry has a commitment to providing preventive education as routine practice in patient treatment. Among oral healthcare personnel, dental hygienists play a central role in providing health education. Dental hygienists are ideal staff for targeting patients who smoke and helping them to stop smoking [4, 5]. Dentists and dental hygienists have been trained in patient education and communication skills that can be easily applied to smoking cessation methodologies. Dental professionals understand that behavioral changes are gradual and require constant reminders. In particular, periodontal therapy offers multiple opportunities for interaction with patients. Because of the negative impact of tobacco use on periodontal treatment, additional motivation for smoking cessation can be demonstrated effectively over time.

For over a decade, oral health professionals have demonstrated a strong interest in and commitment to their involvement in tobacco use intervention programs [6–8]. Numerous controlled studies by oral healthcare professionals have demonstrated the efficacy of health care providers in reducing smoking among their patients [9, 10]. Dental hygienists are regarded as ideal staff for targeting tobacco-using patients, delivering health education messages, and facilitating their patients’ ability to quit smoking.

Tobacco-using healthcare professionals, including dentists, have been reported to be less proactive than their non-using counterparts [11–14]. In the same manner, dental hygienists who smoke may therefore also not be sufficiently proactive when they too are smokers, even thought the Japanese Periodontal Society published a clear tobacco-cessation policy statement in 2003 [15].

In Japan, despite a decline during the 1990s, the rate of smoking among young females has continued to climb steadily since the late 1990s [16]. The smoking prevalence among women is still lower than in western countries, but it has gradually increased to more than 20% of young women. The reason for such an increase among young females is still not well understood. Just as adolescents may begin smoking by modeling the behavior of peers, they may also model the behavior of their parents. Exploring the family background of both smokers and non-smokers may help us to elucidate this phenomenon.

The purpose of this study was to identify the smoking prevalence among dental hygiene students in Japan and to explore the influencing factors in order to clarify the present state regarding the smoking habits of a young female population.

## 2. Materials and Methods

A cross-sectional survey of second-year female oral hygienist school students (the final year of a two-year program) was conducted at a number of dental hygiene schools in Fukuoka Prefecture, Japan from June 2003 to June 2004. All participants were female, because registered dental hygienist courses were restricted to females according to Japanese law at the time of this study. Study personnel not associated with the school managed the survey. The anonymous self-administered questionnaires were distributed and collected by posting. Students who were not willing to participate in the survey were excluded from this study. Approval for the current study was granted by the Fukuoka Dental College Internal Review Board.

The study used a self-administered, forty-eight-item questionnaire that was pilot tested on a small sample of students from other dental hygienist schools. The questionnaire was prepared based on previous surveys of juveniles and healthcare personnel in Japan [17]. Before the study was carried out, these questionnaires were given to dental students not in the study sample as a pilot study and then checked. The collected data were age, smoking status, knowledge regarding the health risk of smoking, participation in smoking prevention programs, and the smoking status of their family members. The questionnaires were administered anonymously during scheduled class times using a standardized introduction.

Frequency distributions were used to describe the data. Bivariate analyses were used to measure any associations between the selected variables, with statistical significance based on the chi-square (χ^2^) test for independence. ANOVA was also used to compare three groups where appropriate. Two-sided tests of significance were based on the 0.05 level against a null hypothesis of no association, unless otherwise indicated. Analyses were mainly performed using SPSS version 11.0J software (SPSS, Chicago, IL).

A classification tree was generated using the package available in the data-mining program Orange [18]. The tree-fitting process initially proceeds by finding the covariate that best divides the subjects into two groups. The best split is defined as the one that results in the most homogeneous subgroups with respect to the response variable. Homogeneity was assessed regarding the misclassification rate at each potential split.

## 3. Results

In total, 168 questionnaires were distributed to female students, of which 164 were returned (97.6% response rate). Six questionnaires were missing answers regarding age or smoking status, which left 158 valid responses that were used for analysis (94.0%). The mean age of participants was 19.8 years (S.D. = 2.1), with ages ranging from 19 to 36.

Individuals who had smoked daily for at least the past six months were classified as current smokers. The percentage of current smokers was found to be 17.1%. Another 3.2% of subjects who reported being smokers were classified as occasional smokers. Among all subjects, 62.9% responded that they had experienced smoking, while 37.1% had never smoked. The mean age of the first smoking experience was 14.9 years old (S.D. = 3.3) for non-smokers, 14.8 years old (S.D. = 1.9) for occasional smokers, and 14.5 years old (S.D. = 2.7) for current smokers, respectively. The age of the first smoking experience showed no statistical difference between groups (F = 0.077, *p* = 0.926). The mean age at which the smoking habit was established among current and occasional smokers was 16.3 years (S.D. = 1.4).

**Figure 1:**
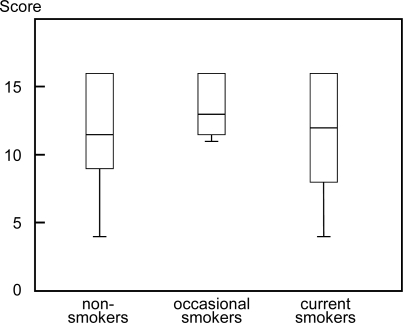
Score of the knowledge of smoking risk in each group. Median scores are indicated by horizontal bars. Vertical bars indicate the range and horizontal boundaries of boxes represent the first and third quartiles. Maximum score was 16 points. ANOVA: F = 0.077, *p* = 0.926.

The score of knowledge on the health risks associated with smoking was calculated by adding the number of correct answers for the questions [17]. The mean knowledge score was 11.8 (S.D. = 3.5) among non-smokers, 13.6 (S.D. = 2.3) in occasional smokers, and 11.6 (S.D. = 3.9) in current smokers, respectively. The knowledge score did not show any statistically significant difference among the groups classified by smoking status (F = 0.683, *p* = 0.506, Figure 1).

While 70.4% of non-smokers had participated in smoking prevention programs, 100% of occasional smokers and 63% of current smokers had taken part in such programs. There was no significant difference among the groups regarding the rate for the participation prevention programs. In addition, 66.7% of current or occasional smokers expressed a desire to quit smoking.

The smoking prevalence of family members showed a significant difference between non-smokers and current or occasional smokers (Table 1). While 11.9% of the mothers of non-smokers were smokers, 68.4% of mothers of current or occasional smokers were smokers (odds ratio = 5.1, 95% confidence interval 2.1–12.2, *p* = 0.000). The smoking rates of other family members, including fathers, did not show any significant statistical difference.

**Figure 2:**
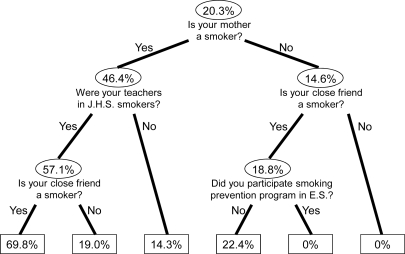
Classification tree analysis of smoking prevalence. Start at the top of the tree. Each ellipse or box is called a “node” or branch point for the tree, whereby further subgrouping was possible. The number in the ellipse is the prevalence of smoking in that group. Boxed values represent terminal nodes containing too few subjects to further subdivide. J.H.S.: junior high school, E.S.: elementary school.

Classification tree analysis thus showed that the smoking habit of dental hygiene students could be well explained by their mother’s smoking history, the smoking status of their junior high school teachers and their close friends, and the experience of having participated in prevention programs while at elementary school (Figure 2).

## 4. Discussion

Similar to other developed countries, the prevalence of smoking is decreasing among the overall population in Japan, while it is increasing among the young female generation in Japan only [16]. This increasing smoking rate in young females is considered to be a serious problem for the control and prevention of smoking in Japan. When the study was conducted in 2003, the smoking rate among Japanese females in their 20s was reported to be 19.2%. Our results among dental hygiene students showed a rate of 20.3% after combining current smokers with occasional smokers, thereby demonstrating almost the same smoking prevalence for the same age group among the general female population in Japan. This result contrasted sharply with a previous study in the U.S. which reported that only 6.9% of dental hygienists were current smokers [4]; therefore, in comparison to western developed countries, Japanese health professionals may lack sufficient awareness of their position as role models for dental patients [14].

Oral healthcare personnel can play an important role in smoking prevention and control. Healthcare professionals can help patients stop smoking by providing them with regular counseling about quitting [19–22]. The phenomenon of health professionals who smoke is therefore considered to have a negative impact on society, since providers who smoke are less proactive in encouraging their own patients to stop smoking [11–14]. In addition, dental hygienists who smoke may also be less likely to counsel current smokers.

In this study, the factors associated with the smoking habit among young female healthcare personnel were examined. The type of residence, the smoking status of family and close friends, the smoking status of school teachers, a history of participating in smoking prevention programs, and knowledge about the risks of smoking were all incorporated into the analysis.

Knowledge about the known health risks associated with smoking may not play an important role in individuals who begin to smoke. There were no differences between smokers and non-smokers regarding the knowledge scores of smoking risks. This phenomenon might reflect the known difficulty of effectively conducting school-based smoking prevention programs [23–25].

The effect of family smoking might be the most important factor for Japanese young females who start to smoke. A previous study reported that family smoking was strongly associated only with the first stage of smoking, though our study showed a significant correlation between an established smoking habit and the mother’s smoking status [25, 26]. The mother’s smoking habit was determined to be the strongest variable affecting smoking in this study. The odds ratio of being a smoker was 5.1 times higher in women with a mother who smoked than in others. Regarding Japanese dental hygiene students, the mother’s smoking habit had a marked influence on her daughter, even if she planned to become a healthcare professional. In addition, smoking by close friends was found to be related to the smoking habit of dental hygiene students, as previously reported [27]. On the other hand, fathers’ smoking status did not affect the smoking habit of their daughters

Another important factor affecting the smoking status of dental hygiene students might be participation in smoking prevention programs. No difference was observed between smokers and non-smokers regarding their age when they first experienced smoking.

The earliest smoking experience among dental hygiene students was reported to be around 6 years of age. Smoking prevention programs can play an important role in the prevention of smoking. Early-stage programs, such as in elementary school, may therefore be needed.

This study has some notable limitations. First, the data were collected by self-report, which could be subject to students’ potential recall bias and a desire to present their behavior in a favorable light; however, since this survey was conducted anonymously, we believe that this phenomenon was negligible. Second, these findings may not be generally valid for individuals from other socioeconomic groups or for other countries.

The important finding of this study was the influence of mothers’ smoking on the smoking habit of their daughters. This means that a mother’s smoking status can thus have a strongly negative influence on her children’s smoking status. It has been reported that adolescents who perceive that both parents would respond negatively and be greatly upset by smoking are thus less likely to smoke [25]. Prevention programs that enhance parental self-efficacy in conveying and enforcing non-smoking policies for their children could thus reduce the rate of smoking among young individuals.

## 5. Conclusions

A relatively high prevalence of smoking by healthcare professionals was identified among dental hygiene students in Fukuoka Prefecture in Japan. Dental hygiene students who had mothers that smoked demonstrated a significantly higher smoking prevalence than others. These results indicate the need to educate dental hygiene students through comprehensive smoking prevention programs. Smoking prevention programs including family members, especially mothers, may be more effective than for individuals alone.

## Figures and Tables

**Table 1:** Percentage of smoking parents among dental hygiene students.

*Prevalence of smokers*	*Smoking status of hygiene students*	
	*Non-smokers*	*Current or occasional smokers*
Father	61.9 % (78/126)	53.1 % (17/32)
Mother	11.9 % (15/126)	40.6 % (13/32)

## References

[1] Rodgers A, Ezzati M, Vander Hoorn S, Lopez A, Lin R, Murray C (2004). Distribution of major health risks: findings from the global burden of disease study. PloS Med.

[2] Ezzati M, Lopez A, Rodgers A, Vander Hoorn S, Murray C (2002). Comparative risk assessment collaborating group. Selected major risk factors and global and regional burden of disease. Lancet.

[3] WHO Regional Office for Europe. Health 21-Health for all in the 21st century: *The health for all policy framework for the WHO European Region. European Health for all Series* **1999**, No.6. Copenhagen. Available at http://www.euro.who.int/document/health21/wa540ga199heeng.pdf (accessed January 15, 2009)

[4] Fried J, Rubinstein L (1990). Attitudes and behaviors of dental hygienists concerning tobacco use. J. Public Health Dent.

[5] Huntley D (1984). What is a dental hygienist?. Dent. Hyg.

[6] Grinstead C, Dolan T (1994). Trends in U.S. dental schools’ curriculum content in tobacco use cessation 1989–93. J. Dent. Educ.

[7] Barker G, Williams K (1999). Tobacco use cessation activities in U.S. dental and dental hygiene student clinics. J. Dent. Educ.

[8] Weaver R, Whittaker L, Valachovic R, Broom A (2002). Tobacco control and prevention efforts in dental education. J. Dent. Educ.

[9] Cohen S, Stookey G, Katz B, Drook C, Christen A (1989). Helping smokers quit: a randomized controlled trial with private practice dentists. J. Am. Dent. Assoc.

[10] Campbell H, Sletten M, Petty T (1999). Patient perceptions of tobacco cessation services in dental offices. J. Am. Dent. Assoc.

[11] Block D, Block L, Hutton S, Johnson K (1999). Tobacco counseling practices of dentists compared to other health care providers in a midwestern region. J. Dent. Educ.

[12] Nardina S, Bertoletti R, Rastelli V, Donner C (1998). Influence of personal tobacco smoking on the clinical practice of Italian chest physicians. Eur. Respir. J.

[13] Sarna L, Brown J, Lillington L (2000). Tobacco-control attitudes, adovocacy and smoking behaviors of oncology nurses. Oncol. Nurs. Forum.

[14] Ohida T, Sakurai H, Mochizuki Y (2001). Smoking prevalence and attitudes toward smoking among Japanese physicians. JAMA.

[15] Japanese Association of Periodontology (2005). Declaration of Opposition to Smoking.

[16] Ministry of Health Law, Japan (2002). The National Health and Nutrition Survey in Japan.

[17] Ohida T, Osaki Y, Okada K, Mochizuki U, Sugie T, Kawahara K, Kawaguchi T, Minowa M (1998). Factors related to smoking habits of students and newly employed nurses. Jpn. J. School Health.

[18] Demsar J Zupan B Leban G Experimental Machine Learning to Interactive Data Mining White Paper Faculty of Computer and Information Science, University of Ljubljana 2004Available at www.ailab.si/orange (accessed November 15, 2008)

[19] Mullen P, Holcomb J, Fasser C (1988). Selected allied health professionals’ self-confidence in health promotion counseling skills and interest in continuing education programs. J. Allied Health.

[20] Secker-Walker R, Hill H, Solomon L, Flynn B (1987). Smoking cessation practices in dental offices. J. Public Health Dent.

[21] O’Shea R, Sielski K, Creola P, Geraci G, Haberer J, Sowinski J (1987). Helping patients quit smoking. The dental hygienist’s role. Dent. Hyg.

[22] Binnie VI, McHugh S, Jenkins W, Borland W, Macpherson LM (2007). A randomised controlled trial of a smoking cessation intervention delivered by dental hygienists: a feasibility study. BMC Oral Health.

[23] Flay B, Koepke D, Thomson S, Santi S, Best J, Brown K (1989). Six-year follow-up of the first Waterloo school smoking prevention trial. Am. J. Public Health.

[24] Murray DM, Perry CL, Griffin G, Harty KC, Jacobs DR, Schmid L, Daly K, Pallonen U (1992). Results from a statewide approach to adolescent tobacco use prevention. Prev. Med.

[25] Jackson C, Henriksen L (1997). Do as I say: parent smoking, antismoking socialization, and smoking onset among children. Addict. Behav.

[26] Madarasová Gecková A, Stewart R, van Dijk JP, Orosová O, Groothoff JW, Post D (2005). Influence of socio-economic status, parents and peers on smoking behaviour of adolescents. Eur. Addict. Res.

[27] Sargent J, Dalton M (2001). Does parental disapproval of smoking prevent adolescents from becoming established smokers?. Pediatrics.

